# Trimineralic abalone shells (*Haliotis iris* Gmelin, 1791) and X-ray diffractometry: A Bayesian calibration model for resolving complex skeletal mineralogy

**DOI:** 10.1371/journal.pone.0346638

**Published:** 2026-04-21

**Authors:** Abigail M. Smith, Peter W. Dillingham, Conor Hassan, Bryce A. Peebles, Ian S. Dixon-Anderson

**Affiliations:** 1 Department of Marine Science, University of Otago, Dunedin, New Zealand; 2 Department of Mathematics and Statistics and Coastal People Southern Skies Centre of Research Excellence, University of Otago, Dunedin, New Zealand; 3 Centre for Data Science, School of Mathematical Sciences, Queensland University of Technology, Brisbane, Queensland, Australia; Argonne National Laboratory, UNITED STATES OF AMERICA

## Abstract

X-ray diffractometry (XRD) is commonly used to determine both aragonite:calcite ratio and Mg content (in calcite) in biogenic skeletal carbonate. Bimineral taxa, such as many abalone, combine aragonite and calcite, or sometimes two distinct calcites, in a single skeleton. At least some abalone shells are, however, formed of three discrete carbonate minerals: aragonite, high-Mg calcite, and low-Mg calcite. Here we develop and apply a new system based on a Bayesian calibration model, an extension of the Reference Intensity Ratio method that accommodates heteroskedastic noise, for determining relative proportions in trimineralic biogenic carbonate using XRD patterns. We describe the system, validate and assess the system using biomineral standards, and quantify sources of error. We then use the system to describe mineralogical variation within the sometimes-trimineralic New Zealand black-footed pāua *Haliotis iris*. All specimens contained aragonite, and most contained low-Mg calcite, with older shell showing decreasing amounts of calcite (presumably due to wear of this external layer). Almost all specimens from Kaikoura contained at least some high-Mg calcite, thus being tri-mineralic. This mixture of three biogenic carbonates is most unusual, so we have used our new method of analysing XRD patterns to estimate the proportions of three co-occurring skeletal carbonate minerals in this marine invertebrate. We also provide the first detailed analysis of uncertainty and precision in XRD analysis of skeletal carbonate mineralogy.

## Introduction

**Fig 1:**
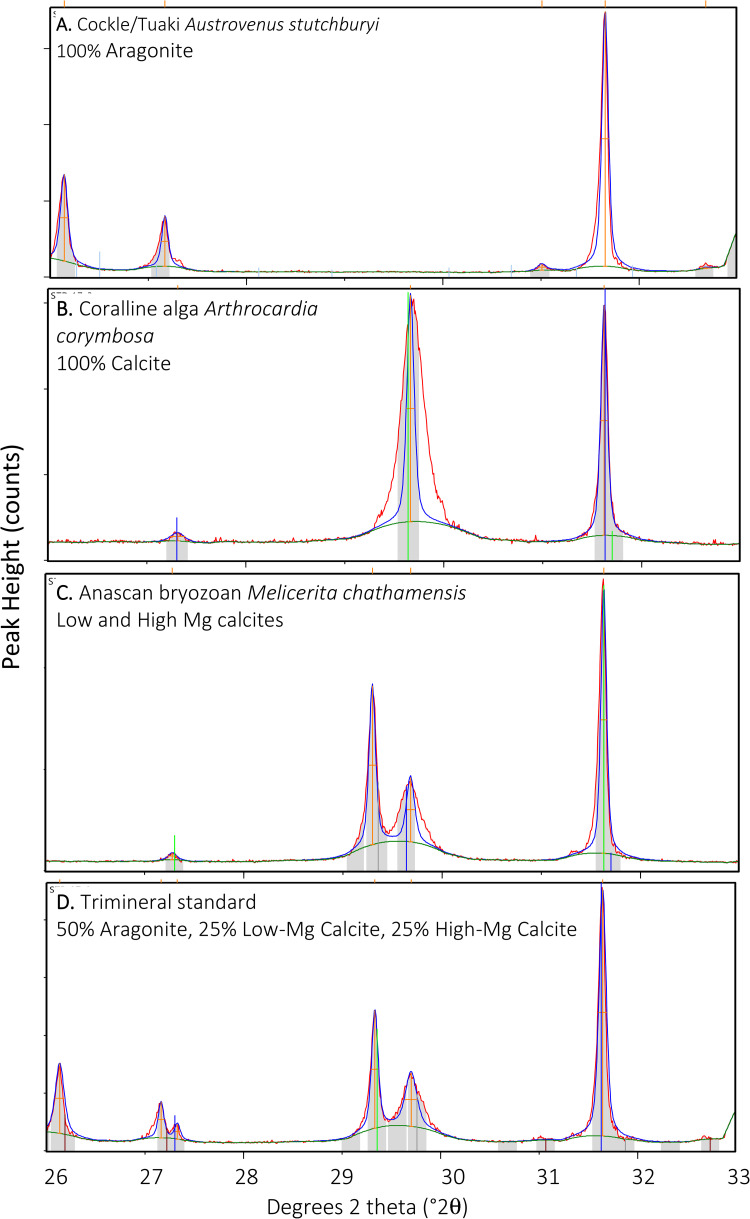
Representative x-ray patterns from marine bryozoan skeletal carbonates. X-axis is the angle of the exiting x-ray beam in degrees 2 theta (°2θ), which varies depending on the crystal structure of the material it encounters. Y-axis is peak height in “counts” which is different for every run and not relevant – it is the relative peak height that indicates proportions of minerals present. Measured peaks are: Aragonite 1 (26.2 °2θ); A2 - Aragonite 2 (27.2 °2θ); C1 - Low-Mg Calcite (29.3 °2θ); C2 - High-Mg Calcite (29.7 °2θ). A small unmeasured calcite peak sometimes appears at 27.5 °2θ. The internal standard of Halite is at 31.7 °2θ.

X-ray diffractometry (XRD) is a common, rapid and inexpensive way to determine the overall carbonate mineralogy of biogenic skeletal material. It has been used a great deal in many taxa since the 1970s to determine both aragonite:calcite ratio and Mg content in calcite. These semi-quantitative techniques rely on interpretation of the location and intensity of a peak on a diffraction pattern by eye or by computer; sometimes both are required (Fig 1). Comparative analysis between labs suggests that calibration of individual XRD machines using standard mixed powders provides the best accuracy and precision [e.g., 1–3].

Acute observers have noted asymmetrical peaks in some XRD patterns of calcite, suggesting that a second peak is located close to the dominant one, and thus that a second mineral is present. Several species of anascan bryozoans in the family Cellariidae, for example, exhibit this dual-calcite mineralogy (Fig 1C). Calibration by standard mixtures has resulted in useful equations allowing for the quantification of proportions of two calcites in species such as *Melicerita chathamens is* [4]. At least some abalone shells are formed of three discrete carbonate minerals: aragonite, high-Mg calcite, and low-Mg calcite. Standard semi-quantitative XRD-based methods for determining relative proportions, however, apply only to a bimineralic biogenic carbonate system, while Rietveld refinement methods [5] rely on specialist software. There is a need to understand skeletal carbonate chemistry and biomineralisation in these important fishery and aquaculture species using widely available tools.

Here we develop a system for analysis of XRD patterns from trimineral biogenic calcareous specimens. Our system is based on an extension of the external standard [6], which forms the basis of the Reference Intensity Ratio (RIR) method of quantitative phase analysis. RIR-based methods can be based on individual peak heights, individual peak areas, or full pattern summation/whole pattern fitting. For complex samples, e.g., soils, the latter, along with the Rietveld method, generally provide better fits [7–9]. The Rietveld method has also been applied to biogenic shell material across a range of Mg concentrations [5]. For relatively simple systems with few peaks and consistent sample preparation, the simplicity and speed of the RIR method can outweigh the accuracy benefits of other methods [9]. Our system uses individual peak heights because a primary goal of the study is to assess fit accuracy and precision under current protocols used by many laboratories in the field, but our approach could be extended to whole-pattern fitting as well (which would have the advantage that it could take into account variable crystallinity). A key feature that we provide is the estimate of uncertainty as well as a point estimate for each weight fraction. In addition, our system can also be used for bimineral biogenic calcareous specimens, i.e., it updates the approaches of others [1,4]. After first validating the system and assessing precision and accuracy, we then use the system to describe mineralogical variation within the sometimes-trimineralic New Zealand black-footed pāua *Haliotis iris*.

## Methods

### Calibration of a trimineral carbonate system

**Fig 2:**
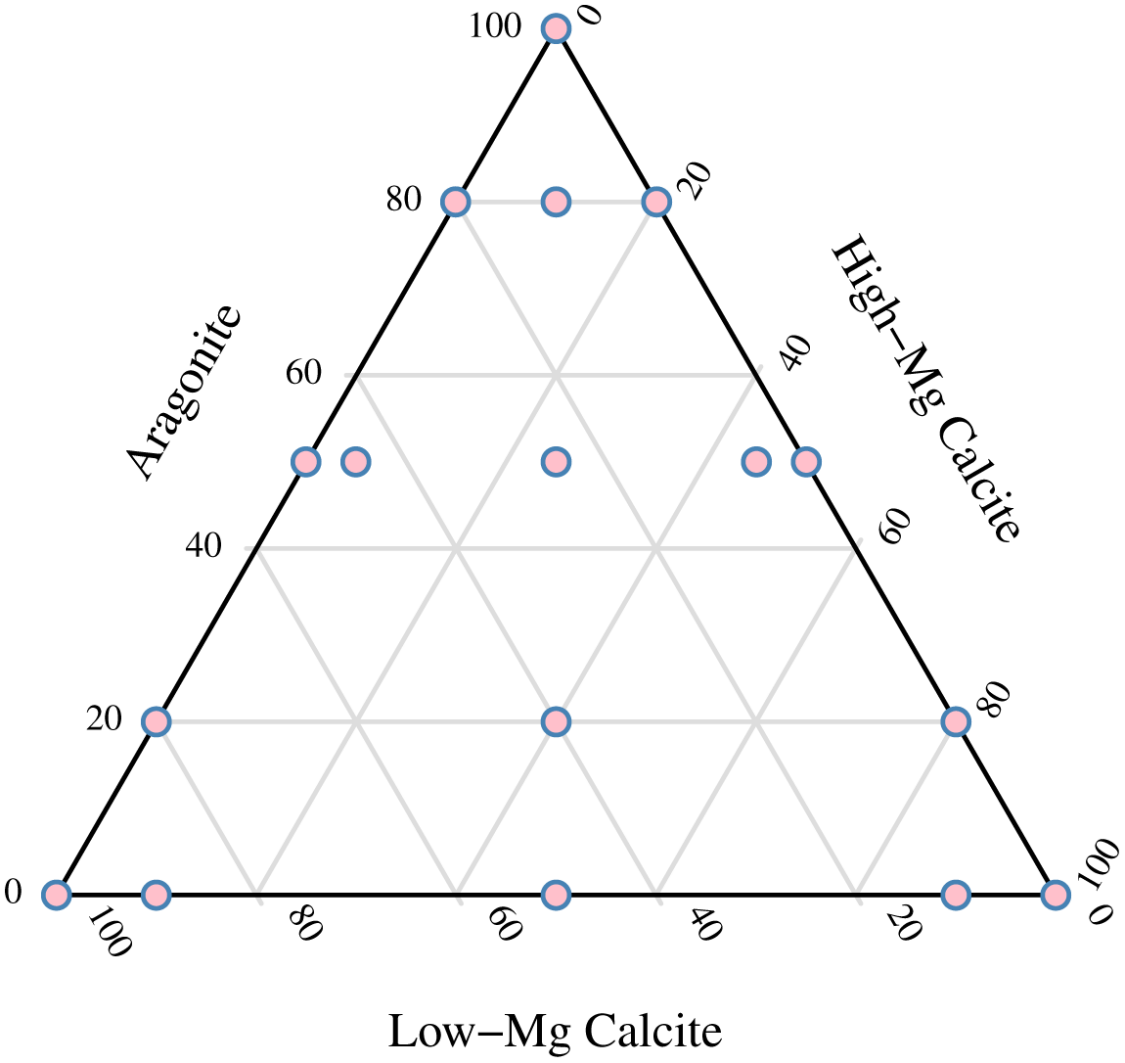
Ternary diagram showing seventeen standards used to calibrate tri-mineralic analysis, consisting of mixtures of Aragonite, high-Mg calcite and low-Mg calcite, in percent by weight.

Standard powders of biogenic carbonate were prepared by obtaining shells from living organisms: aragonite (the New Zealand tuaki/cockle *Austrovenus stutchburyi*), low-Mg calcite (tio/dredge oyster *Ostrea chilensis*: 0.3 wt% MgCO_3_) and high-Mg calcite (erect coralline alga *Arthrocardia corymbosa*: 11.3 wt% MgCO_3_). Shells were manually cleaned and bleached to remove organic material (5% household bleach for 12 hours), then ground in a ring-mill to a fine powder (sieved through a 0.08 mm screen). The powders were analysed by standard XRD methods (see below) 20 times each to ascertain the mean mineralogy of each standard. Seventeen different standard powders were mixed by weight to a total of >5 g in each mixture (Fig 2, Table 1).

**Table 1:** Carbonate mineral composition of 17 standard mixtures used to calibrate trimineralic analysis.

	Percent by Weight		
Sample Number	Aragonite (*Austrovenus stutchburyi*)	Low-Mg Calcite (*Ostrea chilensis*)	High-Mg Calcite (*Arthrocardia corymbosa*)
OU01	100	0	0
OU02	80	0	20
OU03	80	10	10
OU04	80	20	0
OU05	50	0	50
OU06	50	5	45
OU07	50	25	25
OU08	50	45	5
OU09	50	50	0
OU10	20	0	80
OU11	20	40	40
OU12	20	80	0
OU13	0	100	0
OU14	0	10	90
OU15	0	50	50
OU16	0	90	10
OU17	0	0	100

Mixtures were re-ground by hand using an agate mortar and pestle with a few grains of AR-grade halite (NaCl) as an internal standard, thoroughly homogenized, and then three subsamples of 0.3 g each were mixed with ethanol, pressed onto glass slides, flattened with another glass slide to create a uniform and flat surface, and air-dried. Each randomly-oriented specimen was analysed using the X’pert-Pro MPD PW 3040/60 XRD, PANalytical machine at University of Otago, scanned between 26 and 32 °2θ at 50 counts per degree at a count time of 1 sec. Exact peak locations (in °2θ) and heights (in counts from zero) were recorded for peaks identified as Aragonite-1 (26.2 °2θ), Aragonite-2 (27.2 °2θ), Calcite-1 (approx. 29.4 °2θ), Calcite-2 (approx. 29.7 °2θ) and Halite-1 (31.7 °2θ). By examining samples that were pure aragonite, low-Mg calcite, or high-Mg calcite, the Aragonite-2 peak was identified as the half-height shoulder peak of Aragonite-1, Calcite-1 was identified as the primary low-Mg peak, and Calcite-2 was identified as the primary high-Mg peak.

### Current approach to bimineral composition estimation and its limitations

For bimineral compositions, easy-to-use equations have been developed to estimate weight percent given peak height ratios, following the lead of Chave [10] and Milliman [11], among others. Gray and Smith [1], for example, fit a quadratic regression model using Peak Height Ratios (PHR) to estimate calcite:aragonite weight fraction, while Smith and Lawton [4] fit a linear model using calcite peak heights to estimate wt% MgCO_3_ in calcite.

In general, responses from a given XRD instrument/machine have been described using calibration data, where both weight fraction and peak heights/locations were known, and weight fraction was regressed against peak height ratio. This approach is termed ‘inverse’ calibration (Krutchkoff, 1967) because the predictor and response variables are swapped in the regression model. That is, it is the weight fraction of the sample that determines the peak height ratio. The regression model, however, uses peak height ratios to predict weight fractions. While the opposite ‘classical’ calibration [12] is generally recommended, inverse calibration can perform well for simple models and can be implemented using standard tools. Finally, once the XRD output is described by a regression equation, unknown weight fractions can be estimated given observed peak height ratios, e.g., for experimental samples.

These regression approaches are not directly based on a physico-chemical law, but can be justified as polynomial approximations to the nonlinear external standard equation [6]. Limitations include potential bias due to the polynomial approximation, e.g., selecting a linear versus quadratic fit; boundary value problems, where estimated weight fractions can be negative or greater than 100%; and incorrect assumptions around noise, where the regression assumption of constant variability is violated. Consequently, the equations are used to provide useful point estimates, but do not attempt to provide estimates of uncertainty, despite the general importance of doing so when reporting measurement results [13].

When moving to trimineral compositions, numerical complexity increases due to the dimensionality of the data. For bimineral compositions, two peak heights are used to calculate a single peak height ratio, which is then related to a single weight fraction. These are the simplest data to analyse, referred to as zeroth-order [14]. Trimineral compositions, however, must link a vector of peak height ratios to a vector of weight fractions that are constrained to sum to 1. For higher-order data like this, many standard techniques do not perform well. Particularly, the inverse calibration method that can be used effectively for zeroth-order data is not recommended for higher-order nonlinear systems [15]; point estimates may be reasonable, but uncertainty estimates rarely are. To accommodate higher-order data and to overcome the limitations of the current equations, we developed a Bayesian system that directly employs the external standard equation, can be used for bimineral or trimineral compositions, and could be extended to more complex settings such as full pattern summation data.

### Development of a system for trimineral composition estimation

We developed a two-component system that allows us to convert data from an XRD pattern into estimated mineral proportions by weight. The components (1) describe the instrument using mixtures of known composition and then, (2) for new samples with unknown composition, estimate the weight percent for each mineral using peaks heights derived from XRD patterns. In addition to point estimates of mineral proportions by weight, our approach also allows us to estimate uncertainties in the weight proportions. Both components are based on the external standard equation [6]. The equation links mineral proportions by weight *(X)* to peak intensities (*I*) via reference intensity ratios (*k*). We add an additional parameter (κ) to allow for noise.

The first component is a Bayesian calibration model that uses mixtures of known composition to describe the instrument and estimate RIRs, and run via the statistical programming language R [16] accessing the program JAGS [17] via the rjags package [18]. This component needs to be run periodically to accommodate potential machine drift, or run if there is a change in laboratory practices, e.g., in sample preparation. Because JAGS is accessed via R, no prior knowledge of JAGS or Bayesian methods is required; the only interaction with the freely available, cross-platform JAGS software is its initial installation. It requires some knowledge of R to run, but the code is incorporated within an R script making it widely accessible to anyone with basic knowledge of R.

The second component is used to estimate the mineral weight proportions for new samples with unknown composition based on the observed patterns. It is accessed via R or via an easy-to-use Excel spreadsheet, where output from the first component is entered and can be updated as needed. For routine calculations, it only requires entry of peak heights for the three minerals, with all other calculations performed automatically. Bimineralic compositions are accommodated by setting peak heights to 0 or NA for any mineral that does not qualitatively appear in the XRD pattern. As this second component will be used most often and by the widest number of people, providing an implementation via Excel reduces the technical level required to use the system within a given laboratory.

In addition to technical details given below, software and the example dataset are publicly available [19].Briefly, while the underlying model that we use is based on the nonlinear external standard equation [6], we extend it by treating the observed peak heights as noisy realizations of expected intensities, recognizing that two runs from the same underlying sample prepared using the same protocols will produce similar but not identical results. Particularly, we model noise as a heteroskedastic process that depends on peak height and sample proportion. The second component uses the multivariate version of the delta method [20] to estimate variability due to the instrument and laboratory practices, and uncertainty due to imprecision in the calibration parameters, i.e., the reference intensity ratios and the noise parameter. Typically, uncertainty from imprecision in the calibration parameters is substantially lower than the other sources of variability.

We then applied our system to the 17 mixtures of known composition. As described previously, each mixture was divided into three subsamples, with a separate XRD analysis for each subsample. We randomly selected one subsample from each mixture as inputs for the first system component, and then used those calibration results to predict the composition of the other two subsamples using the second system component. Specifically, this allowed us to evaluate in-sample system performance by examining empirical error for each of the remaining 34 subsamples based on the *n =* 17 sample calibration dataset.

We also compared our method to the high-Mg-Calcite regression equation from Smith and Lawton [4]. For this, we used the 32 subsamples from the *n* = 16 samples that had calcite present. We then estimated the proportion of calcite that was high-Mg calcite using both methods, and compared the estimates to each other to assess whether the new approach led to a substantive improvement.

### Detailed structure of the model

The external standard equation for a trimineral composition, derived from [6], is:


$${\hat{\pi }}_{l}=\frac{k_{m}k_{n}I_{l}}{k_{\text{2}}k_{\text{3}}I_{\text{1}}+k_{\text{1}}k_{\text{3}}I_{\text{2}}+k_{\text{1}}k_{\text{2}}I_{\text{3}}}\left(=\frac{\frac{I_{l}}{k_{l}}}{\sum_{i=\text{1}}^{\text{3}}\frac{I_{i}}{k_{i}}}\right),$$

where ${\hat{\pi }}_{l}$ is the estimated weight fraction of mineral *l* given integrated intensities or peak heights *I*_1_, *I*_2_, and *I*_3_ for the three minerals with reference intensity ratios *k*_1_, *k*_2_, *k*_3_. The reference intensity ratios can be defined re*l*ative to an external standard, or estimated relative to one of the three minerals, e.g., by setting *k*_1_ = 1 and then *k*_2_, *k*_3_ are intensity ratios relative to the first mineral.

If the only purpose of the model was to estimate reference intensity ratios and weight fractions, a classical least squares approach could be taken with minimal differences. However, a model for noise in peaks heights is required to quantify uncertainty in weight fractions. Due to the hierarchical nature of the data, a Bayesian approach was the natural choice.

We start with a generative model for observed intensities, which is used to build a calibration model. The observed intensity for the l^th^ mineral is assumed to come from a normal distribution centered on the expected intensity ($\lambda_{l}$), with variance proportional to the mean:


$$I_{l}\sim N\left(\text{mean}{=\lambda }_{l},\text{variance}=\kappa \lambda_{l}\right),$$

where κ is a machine- and practice-dependent noise parameter. Then, letting $\Lambda =\sum_{i=\text{1}}^{\text{3}}\lambda_{i}$, we can calculate the expected relative intensities ($r_{l}$) as


$$E\left(r_{l}\right)=\frac{\lambda_{l}}{\Lambda }=\frac{k_{l}\pi_{l}}{\sum_{i=\text{1}}^{\text{3}}k_{i}\pi_{i}},$$

where $\pi_{i}$ is the actual weight fraction of mineral i in the trimineral composition. For our model and software, we treat the first mineral as the standard and set $k_{\text{1}}=\text{1}$ without loss of generality. Priors for the Bayesian model were Λ~Gamma(0.001,0.001), and $k_{\text{2}},k_{\text{3}}\sim \text{Uniform}(\text{0}.\text{01},\text{100})$. Model fit was assessed using Bayesian *p*-values [21] for three summary statistics (one for each mineral), where each was the sum of squared residuals scaled by its standard deviation. Models were run in JAGS [17] via the rjags package [18] implemented in R v. 4.3.1 [16].

The first component of the system, the Bayesian calibration model, combines the generative model above with mixtures of known composition to estimate the reference intensity rations (*k*_2_, *k*_3_), their variances ($\overset{\text{2}}{\underset{k_{\text{2}}}{\sigma }},\overset{\text{2}}{\underset{k_{\text{3}}}{\sigma }}$), covariance ($\sigma_{k_{\text{2}}k_{\text{3}}}$), and the noise parameter κ*.*

Once this step is complete, the second component provides an estimate of each weight fraction (${\hat{\pi }}_{l},\;l\in \{\text{1},\text{2},\text{3}\}$) based on Equation 1 using measured peak heights or intensities and the estimated reference intensity ratios (${\hat{k}}_{\text{2}},\;{\hat{k}}_{\text{3}}$). Uncertainty in ${\hat{\pi }}_{l}$ is estimated using the multivariate version of the delta method [20]. There are two sources of noise that drive uncertainty: inherent variability due to the instrument and laboratory practices ($\sigma_{I}$), and uncertainty due to imprecision in the calibration parameters ($\sigma_{C}$), where the combined uncertainty is $\sigma_{{\hat{\pi }}_{l}}=\sqrt{\overset{\text{2}}{\underset{I}{\sigma }}+\overset{\text{2}}{\underset{C}{\sigma }}}$. The source of noise due to inherent variability for mineral l is estimated by:


$$\overset{\text{2}}{\underset{I,l}{\hat{\sigma }}}=\overset{\text{2}}{\underset{l}{\phi }}\frac{{\hat{k}}_{m}{\hat{k}}_{n}{\hat{\pi }}_{l}{\left(\text{1}-{\hat{\pi }}_{l}\right)}^{\text{2}}+{\hat{k}}_{l}\overset{\text{2}}{\underset{l}{\hat{\pi }}}({\hat{k}}_{m}{\hat{\pi }}_{n}+{\hat{k}}_{n}{\hat{\pi }}_{m})}{{\hat{k}}_{m}{\hat{k}}_{n}(\text{1}-\;{\hat{\pi }}_{l})},$$

where:


$$\overset{\text{2}}{\underset{l}{\phi }}=\hat{\kappa }\frac{{\hat{\pi }}_{l}(\text{1}-{\hat{\pi }}_{l})}{I_{l}}.$$

The second source of noise due to uncertainty due to imprecision in calibration parameters for mineral l is estimated by:


$$\overset{\text{2}}{\underset{C,l}{\hat{\sigma }}}=\overset{\text{2}}{\underset{k_{\text{2}}}{\hat{\sigma }}}{\left(\frac{\partial {\hat{\pi }}_{l}}{\partial k_{\text{2}}}\right)}^{\text{2}}+\overset{\text{2}}{\underset{k_{\text{3}}}{\hat{\sigma }}}{\left(\frac{\partial {\hat{\pi }}_{l}}{\partial k_{\text{3}}}\right)}^{\text{2}}+\text{2}{\hat{\sigma }}_{k_{\text{2}}k_{\text{3}}}\frac{\partial {\hat{\pi }}_{l}}{\partial k_{\text{2}}}\;\frac{\partial {\hat{\pi }}_{l}}{\partial k_{\text{3}}},$$

where $\frac{\partial {\hat{\pi }}_{l}}{\partial k_{l}}=\frac{-k_{m}k_{n}I_{l}(k_{m}I_{n}+k_{n}I_{m})}{{\left(k_{\text{2}}k_{\text{3}}I_{\text{1}}+k_{\text{1}}k_{\text{3}}I_{\text{2}}+k_{\text{1}}k_{\text{2}}I_{\text{3}}\right)}^{\text{2}}\;}$ and $\frac{\partial {\hat{\pi }}_{l}}{\partial k_{m}}=\frac{k_{l}\overset{\text{2}}{\underset{n}{k}}I_{l}I_{m}}{{\left(k_{\text{2}}k_{\text{3}}I_{\text{1}}+k_{\text{1}}k_{\text{3}}I_{\text{2}}+k_{\text{1}}k_{\text{2}}I_{\text{3}}\right)}^{\text{2}}\;}$; use of estimated rather than actual ks has minimal effect onestimates of $\frac{\partial {\hat{\pi }}_{l}}{\partial k}$. For a sufficiently accurate calibration routine, the first source of noise should dominate the second source. If not, that would suggest the need for more calibration data.

Note that a Bayesian approach to the second component could also be developed. However, the choice of the delta method provided several advantages: (a) no need for modularization of a combined model to keep priors for experimental data from compromising the calibration routine [18,22], (b) no need to re-run the calibration calculations each time a new set of experimental data are acquired, and (c) the ability to implement the second model in either R or an Excel spreadsheet.

#### Reproducibility (precision and accuracy).

To evaluate the precision of X-Ray Diffractometry of powdered trimineral carbonate, a single powdered specimen composed of 50% aragonite, 25% low-Mg calcite and 25% high-Mg calcite was analysed 20 times, all in the same afternoon, without being moved from the XRD chamber, in order to estimate ‘instrument’ precision. The same specimen was then analysed 20 times, having been loaded and unloaded into the diffractometer each time, on several different days, thus adding an ‘operator’ component to the precision. Finally, 20 different subsamples from the same homogenized sample of mixed powder were analysed (all on the same day), adding a ‘sampling’ component to the overall precision.

#### Mineralogy of *Haliotis iris.*

Specimens of *Haliotis iris* (including as wide a size range as possible) were collected from aquaculture farms, museum collections, and subtidal kelp forests at Karitane and at Kaikoura, both on the east coast of the South Island, Aotearoa New Zealand. In total 29 specimens were sampled, from latitudes 40.9 to 45.5 °S, and ranging from 22 to 135 mm in length (Table 2).

**Table 2:** Locality data for specimens of New Zealand black-footed pāua *Haliotis iris* used in this study.

Sample number	Location	Latitude	Longitude	Collector(s)	Shell length (mm)
HI-CB-72	Coppermine Bay, Marlborough Sounds	−40.932	173.798	Lakeman	72
HI-CR-80	Croiselles Bay, Nelson	−41.065	173.662	Lakeman	80
HI-MB-88	Mahanga Bay Aquaculture, Wellington	−41.292	174.834	Walton	88
HI-MB-94	Mahanga Bay Aquaculture, Wellington	−41.292	174.834	Lakeman	94
HI-MB-38	Mahanga Bay Aquaculture, Wellington	−41.292	174.834	Lakeman	38
HI-KK-30	Kaikoura, South Island	−42.399	173.679	Rayment	30
HI-KK-37	Kaikoura, South Island	−42.399	173.679	Rayment	37
HI-KK-42	Kaikoura, South Island	−42.399	173.679	Rayment	42
HI-KK-56	Kaikoura, South Island	−42.399	173.679	Rayment	56
HI-KK-65	Kaikoura, South Island	−42.399	173.679	Rayment	65
HI-KK-71	Kaikoura, South Island	−42.399	173.679	Rayment	71
HI-KK-87	Kaikoura, South Island	−42.399	173.679	Rayment	87
HI-KK-112	Kaikoura, South Island	−42.399	173.679	Rayment	112
HI-KK-135	Kaikoura, South Island	−42.399	173.679	Rayment	135
HI-KC-119	Kaingaroa, Chatham Is	−43.742	−176.286	Smith & Spencer	119
HI-KC-86	Kaingaroa, Chatham Is	−43.742	−176.286	Smith & Spencer	86
HI-KC-50	Kaingaroa, Chatham Is	−43.742	−176.286	Smith & Spencer	50
HI-SNZ-22	Karitane, South Island	−45.65	170.667	Hepburn	22
HI-SNZ-24	Karitane, South Island	−45.65	170.667	Hepburn	24
HI-SNZ-32	Karitane, South Island	−45.65	170.667	Hepburn	32
HI-SNZ-37	Karitane, South Island	−45.65	170.667	Hepburn	37
HI-SNZ-39	Karitane, South Island	−45.65	170.667	Hepburn	39
HI-SNZ-61	Karitane, South Island	−45.65	170.667	Hepburn	61
HI-SNZ-80	Karitane, South Island	−45.65	170.667	Hepburn	80
HI-SNZ-81	Karitane, South Island	−45.65	170.667	Hepburn	81
HI-SNZ-87	Karitane, South Island	−45.65	170.667	Hepburn	87
HI-SNZ-88	Karitane, South Island	−45.65	170.667	Hepburn	88
HI-SNZ-109	Karitane, South Island	−45.65	170.667	Hepburn	109
HI-K	Karitane, South Island	−45.65	170.667	Hepburn	--

**Fig 3:**
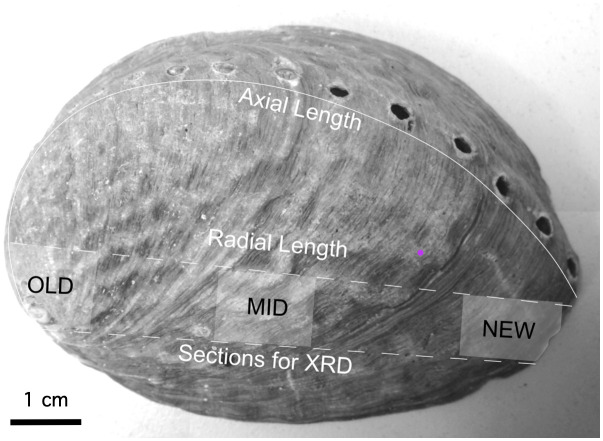
Pāua shell with areas selected for XRD analysis highlighted, including: axial length (solid white line), and radial length (top dashed line), and where shells were sectioned (along the dashed lines). The spire (OLD), middle (MID), and edge (NEW) sections were powdered for XRD analysis.

Each shell was photographed and shell length was measured: axial length (AL) from umbo to edge along the curve of the shell, and radial length (RL) in a straight line (Fig 3). Shells were cut in half along the central axis, and one half saved for further geochemical analysis. The remaining shell was cut into “old”, “middle”, and “new” sections (see Fig 3) before grinding into powder. Three replicates of each powder (thus, for each shell *n* = 9) were analysed using the X-ray diffraction method described above. Patterns with ragged peaks or poorly-resolved peak heights were discarded, so that there are 252 samples in the final analysis from 28 specimens.

## Results

### A new system for determining composition of bimineral and trimineral mixtures

The 17 samples of known composition were used to estimate the model parameters for the first component of the new system: two reference intensity ratios (*k*_2_, *k*_3_ for low-Mg calcite and high Mg-calcite, measured relative to aragonite), their variances and covariance ($\overset{\text{2}}{\underset{k_{\text{2}}}{\sigma }},\;\overset{\text{2}}{\underset{k_{\text{3}}}{\sigma }},\;\sigma_{k_{\text{2}}k_{\text{3}}}$) and the noise parameter (κ). Using 500,000 Markov chain Monte Carlo iterations, parameter estimates were ${\hat{k}}_{\text{2}}=\text{3}.\text{5}\text{1}$ (95% credible interval: 2.97–4.19); ${\hat{k}}_{\text{3}}=\text{1}.\text{4}\text{9}$ (1.22–1.83); $\hat{\kappa }=\text{4}\text{3}.\text{1}$ (19.5–93.4); with estimated standard deviations $\overset{\text{2}}{\underset{k_{\text{2}}}{\hat{\sigma }}}=\text{0}.\text{0}\text{9}\text{4};\;\overset{\text{2}}{\underset{k_{\text{3}}}{\hat{\sigma }}}=\text{0}.\text{0}\text{2}\text{3};{\hat{\sigma }}_{k_{\text{2}}k_{\text{3}}}=\text{0}.\text{0}\text{2}\text{4}$. Both numerical and model diagnostics were good, based on visual inspection of traceplots, Brooks-Gelman-Rubin diagnostics of 1, and Bayesian *p*-values for three diagnostics equal to 0.50±0.05. We emphasize that different estimates would be expected for a different machine or the same machine with different laboratory practices. That is, parameter estimates should be treated as machine- and laboratory practice-specific, and a calibration procedure should be run prior to using the second component of the system to estimate new samples.

**Fig 4:**
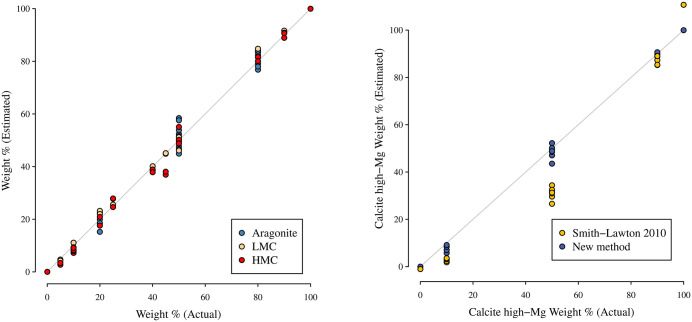
Calibration Results. **A.** Estimated weight fractions are aligned with actual weight fractions for all three minerals (*R*^*2*^ > 0.99 for all minerals; estimated slopes = 1.01, 1.01, 1.00 for Aragonite, Low-Mg Calcite (LMC) and High-Mg Calcite (HMC)). **B.** Comparison of calibration from [4] using standard bimineral PHR correlation and the calibration from this study shows the improvement from this study for analysing calcite subcompositions.

**Fig 5:**
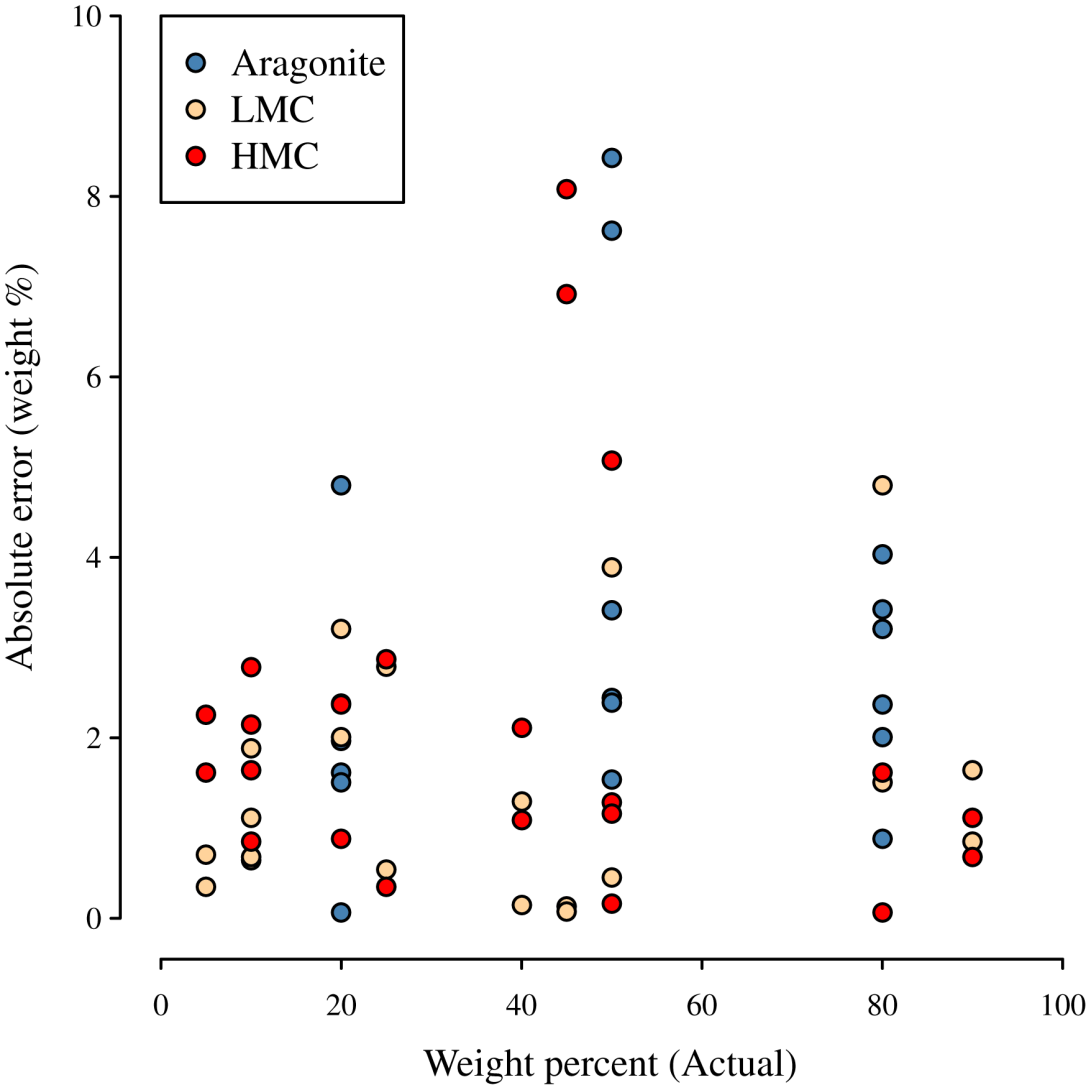
The mean absolute error across all samples with non-zero weight fractions was 2%. However, the error depends on the weight fraction, and is highest when the weight fraction equals 50% (for a given peak height). Error also depends on the sum of peak heights, decreasing as the sum increases (not shown). Minerals: Aragonite, Low-Mg Calcite (LMC) and High-Mg Calcite (HMC).

The second component used the values above and measured peak heights to estimate the composition of 34 subsamples from the same 17 mixtures. Because the composition of these subsamples was known, we used them to compare the estimated weight fraction to the actual weight fraction (Fig 4a). Strong alignment between actual and predicted weight fractions was observed throughout (*R*^2^ > 0.99, regression slopes ≈ 1) (Fig 4a). For the 16 mixtures with calcite present, there was clear improvement using the new method versus the regression equation from Smith and Lawton (2010) for estimating calcite composition (Fig 4b), demonstrating the utility of the new method for bimineral compositions as well as trimineral compositions. Particularly, the equation from [4] yields substantively biased estimates, whereas the new method does not. For the trimineral compositions, the overall mean absolute error was 2% (excluding pure samples where error is 0 by default); it does, however, depend on the sample composition and peak heights. Absolute error is smaller when the weight fraction is near 0 or 1, and larger when it is near 50% (Fig 5).

Errors are due to a variety of factors, including instrument, operator, and sample variability, which may be reduced by having a better machine and improved laboratory practices, but never eliminated entirely (intrinsic errors). Errors are also due to noise in the calibration process (calibration error), which can be reduced by increasing the number of calibration samples. The second component of the system provides estimates of the overall standard error, as well as intrinsic and calibration components, for each sample. On average, uncertainty from the calibration procedure added 5% to the standard error, relative to the intrinsic error alone. E.g., a sample with intrinsic standard error of 4.0% (absolute) might increase to 4.2% (absolute) combined standard error due to uncertainty in the calibration parameters. This result suggests that the calibration procedure had sufficient precision, and increasing the number of calibration points would have limited benefit.

### Precision and accuracy of XRD analysis of biogenic carbonate mixtures

**Fig 6:**
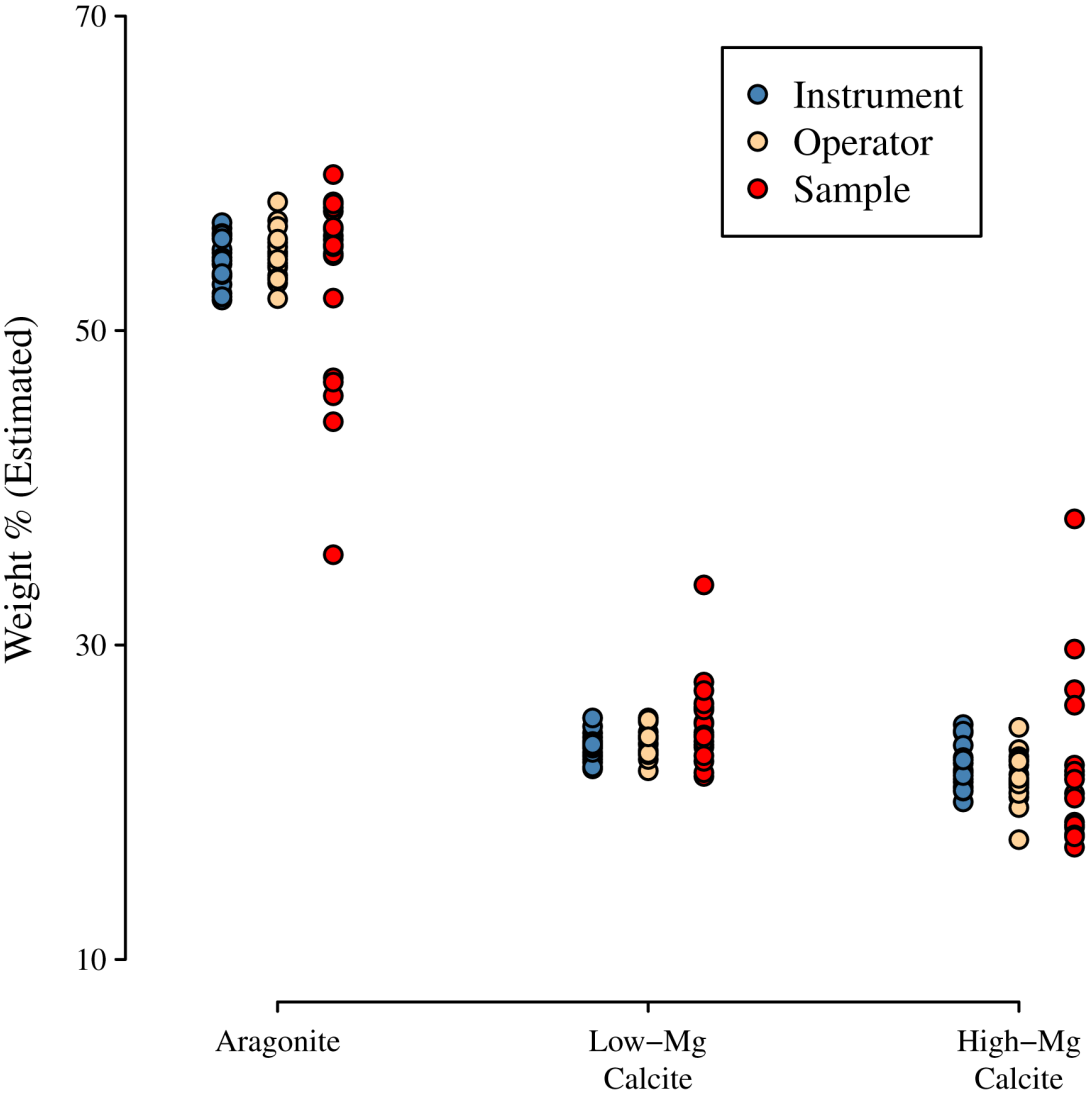
Precision for repeated measurements of a single trimineral sample were similar whether the sample remained in the instrument, or was removed and replaced by an operator (with no change to the smear mount). Measurements of different samples from the same underlying mixture showed greater variability due to random assortment.

Three different approaches were used to characterise precision in this process, examining instrument error, operator error, and sampling error. Raw peak heights across all minerals varied greatly (ranges of 452–3554 counts, *n* = 60), as they are partially a function of total sample size and orientation. In general, however, compositional analysis relies on relative peak heights, though the estimation technique is more precise when peak heights are greater (raw data available in Dillingham et al., 2026). When converted into estimated compositions, the instrument and operator precisions were similar (standard deviations of 1% absolute for all three components). However, variability increased substantially when sample variability was included (standard deviations 6%, 3%, and 5% absolute for aragonite, high-Mg calcite, and low-Mg calcite; Fig 6).

### Mineralogy of *Haliotis iris*

**Fig 7:**
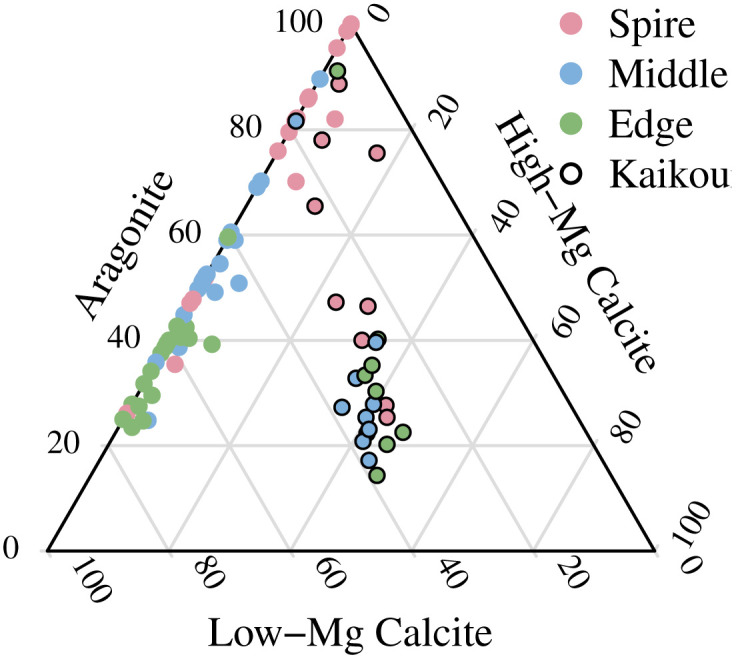
Skeletal carbonate mineralogy of 252 samples from 28 individual specimens of *Haliotis iris*, mostly from southern New Zealand. All samples contain aragonite, 96% contain low-Mg calcite, and 39% contain both high-Mg calcite and low-Mg calcite.

All our specimens of *Haliotis iris* contained aragonite, with measurements ranging from 12 to 100 wt% (mean = 49.9 wt%, *n* = 252 measurements of 28 individuals, SD = 24.2 wt% aragonite). Almost all specimens contained low-Mg calcite (LMC), with only eight measurements from three shells lacking LMC; one of the three shells had two measurements where no discernible LMC peak was visible and one measurement estimated to be 0.1% LMC. All of these low-to-no LMC were all from the spire section of large shells. Only 99 measurements showed any HMC, mostly in the specimens from Kaikoura (8 of 9 shells and 69 of 81 measurements; sample numbers beginning HI-KK, Fig 7). Over the full collection of 252 measurements from 28 specimens of *Haliotis iris* (raw data available in [19]), the average mineral composition was 50% aragonite, 40% low-Mg calcite (LMC) and 10% high-Mg calcite (HMC). Because mineralogy varies by location, these values should only be considered as descriptions of this dataset, and cannot be used to make inferences about *Haliotis iris* shells more broadly.

### Patterns in skeletal carbonate mineralogy of *Haliotis iris*

Mineralogy was examined across 28 shells at three shell locations corresponding to shell age, with three subsamples taken per location, for a total of 252 measurements. The three subsamples at each location on each shell were averaged together for further analysis, leading to 84 averaged measurements for the 28 shells corresponding to shell age (the edge, middle, and spine of the shell). Mixed models were run to estimate the effect of shell age, sampling location, and shell length, with a random shell effect included to avoid pseudoreplication, run via the lme4 package in R [23]. One shell did not have a length measurement, so 81 measurements from 27 shells were included in these analyses. Marginal means, or means adjusted for differences in shell lengths and number of shell at the various sampling locations, were estimated using the emmeans package in R [24]. The variation explained by the three fixed effects was estimated via conditional *R*^2^ for combined effects [25] and part *R*^2^ for fixed effects [26]. For aragonite and LMC, linear mixed effects models were run; for HMC, a linear mixed effects model was run for those samples where HMC was present, complemented by descriptive statistics of the pattern of presence/absence. We note that the locations we sampled were not randomly selected, so the estimates below are only valid across those locations, and we further assume that the sampled specimens are suitably representative of their locations.

On average, shell spires were dominated by aragonite at 67% (95% CI: 60–75%) and 33% calcite. The calcite was generally made up of LMC (95% CI: 21–33%); few spire samples contained HMC. In contrast, carbonate from the middle of the shell was approximately evenly split between aragonite and LMC, on average 46% aragonite (95% CI: 39–54%) and 44% LMC (95% CI: 38–50%). Young shell edges were on average 35% aragonite (95% CI: 28–42%), with on average 54% LMC (95% CI: 48–61%). HMC appeared in only a few samples, and those were almost all in Kaikoura (see below).

For HMC, we first examined presence versus absence. In specimens from Kaikoura, 26 of 27 (averaged) samples had HMC present, ranging from 2% to 47%. Across the other locations, HMC was uncommon (15 of 54 samples) and at low levels when present (1–6%). In Kaikoura, similar amounts were found in the edge (37%; 32–42%) and middle (38%; 33–44%) of the shell, with somewhat reduced amounts in the spire (26%, 21–31%). At other locations, average HMC levels were low across the shell, with average values between 0 and 4%.

Shell length in pāua is strongly correlated with age [27]. On average, aragonite percentage increases with size, with a 10 mm increase in shell length corresponding to a 2.2% (0.8–3.6%) increase in aragonite and a corresponding decrease in calcite.

**Fig 8:**
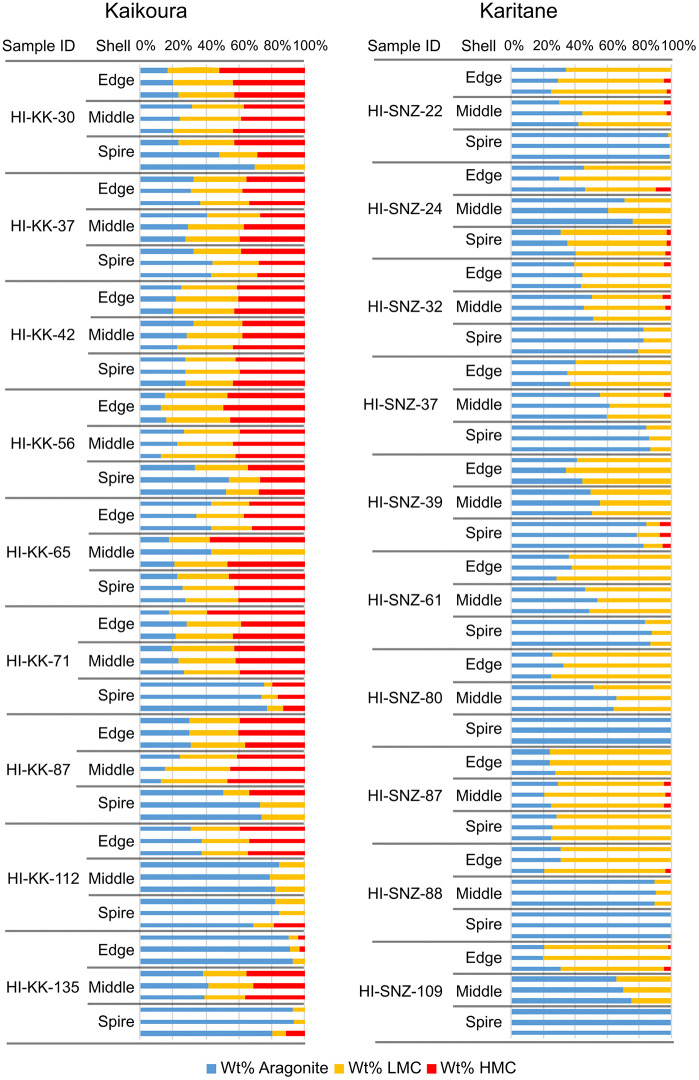
Skeletal carbonate mineralogy of individual specimens of *Haliotis iris* from Kaikoura (left) and Karitane (right). Specimen names indicate species (HI) – location (KK or SNZ) – shell length in mm (from 22 to 135 mm) – part of the shell sampled (spire, middle, edge, see Fig 3) – and replicate (*n* = 3).

We acquired two growth series: nine shells ranging from 30 to 135 mm in length from Kaikoura (northeastern South Island, latitude −42.4) and eleven shells ranging from 22 to 109 mm from Karitane (southeastern South Island, latitude −45.7) (Fig 8). As previously noted, there were large differences observed for HMC in Kaikoura. Kaikoura also had correspondingly lower average levels of aragonite (40%; 32–48%) compared to the location with the highest estimated levels (Karitane, 59%; 51–66%). Other locations averaged near 50% aragonite but were not clearly differentiated from either Kaikoura or Karitane. Similarly, LMC was lower in Kaikoura (28%, 22–34%) than other locations, where average LMC was estimated from 42 to 51%, with lower limits of 95% confidence intervals all above 35%. None of the other locations were clearly differentiated from each other.

The location of the two calcite minerals in the shell is not clear. We attempted Raman spectroscopy [28], microprobe analysis, and SEM-EDS/IBSD analyses, as well as mineral staining, but none of them were sufficiently sensitive to discern between LMC and HMC on the scale of a pāua shell cross-section. The use of XRD at least identifies the minerals present, though it can do little to pinpoint location.

## Discussion and conclusions

### A new trimineral skeletal carbonate model

We have developed a new method that uses data from XRD patterns to estimate the proportions of three co-occurring skeletal carbonate minerals. While the model is more complex than most carbonate sedimentologists are used to, a readily-available Excel spreadsheet allows for straightforward estimations to be made from peak heights. A new feature is the capacity for users to understand and report on the uncertainty in their data.

The two component-system comprises a calibration model and an estimation model. The calibration model extends the external standard equation [6], using samples of known composition to estimate model parameters, including parameters that allow us to estimate uncertainty. This calibration is a Bayesian model that is accessed via R. It is not difficult to run for R users, and only needs to be run occasionally. We then enter the estimated parameters from the first component into an Excel spreadsheet (the second component) to estimate compositions and standard errors for samples of unknown composition. This estimation model provides greater access for research groups: only one reasonably competent R user is required to install and run the workflow to perform the instrument-specific calibration, with no need for specialist Bayesian expertise.

When fitting the first component to 17 calibration samples and assessing fit versus 34 additional samples using the second component, we found good accuracy (see Fig 4a) and outperformance of earlier regression-based approximations [1,4] (see Fig 4b). This is the first instance we are aware of where standard errors are estimated from the external standard equation. We note that these model-based standard errors were somewhat but not overly conservative, e.g., all observed errors fell within ±2 standard errors and 89% within ±1 standard errors.

There are three possible extensions to our model. First, if there was a desire to assess whether a mineral is conclusively in the sample (versus a signal consistent with 0), measured intensities would need to be included for all peaks, even those that qualitatively appear to not exist. However, this process would necessitate adding a background noise term to the variability in intensity and determining the limit of detection for the non-linear estimator for weight fraction ([29]. Next, the model could be extended to include secondary peaks for a given mineral. However, there did not appear to be any substantive advantage to doing so in the dataset we examined, and we therefore opted for simplicity by only incorporating dominant peaks for each mineral. Finally, the model could be modified to accommodate more complex sampling schemes, e.g., for analysis of non-independent sub-samples.

### Extensions to other systems and current limitations

The framework could extend to systems with more than three minerals, provided calibrated RIR values are available and the diffraction pattern contains sufficient independent information to distinguish among minerals. In practice, this approach requires adequate peak resolution to ensure identifiability; the selected angular range should include at least one well-resolved, non-coincident peak per mineral present. Performance will deteriorate when peak intensities cannot be reliably attributed to specific minerals or when substantial peak overlap induces confounding among components. The underlying software could be modified for full pattern summation to account for this, but substantial work would be required to reproduce the ease-of-use of the current Excel spreadsheet. Likely, a fully Bayesian workflow in R would be used instead. In settings where contamination, unmodelled and minor crystalline components, or amorphous material affect peak intensities, bias would occur unless data are corrected or the model extended to specifically account for them, e.g., by subtracting a background signal from each peak. Because RIRs are determined empirically for a given system and experimental protocol, they are robust within that context. While variable crystallinity can lead to peak broadening, so long as the height is proportional to total intensity for a given peak, the constant of proportionality will be absorbed into the RIR; the proportionality constant need not be the same from one peak to another.

Two assumptions for our system warrant further evaluation. First, RIR values estimated from single-phase biogenic standards are assumed to be transferable across taxa expressing the same mineral species. Standards of low-Mg calcite, high-Mg calcite, and aragonite were selected for mineralogical purity and analytical convenience, and applied to abalone shells. We thus assume that mineral-specific differences in texture, compositional substitution, or microstructure do not substantially alter relative peak intensities at a given Mg concentration. If such differences were present, organism-specific validation or recalibration would be appropriate.

Additionally, the specific composition of low- and high-Mg calcite may influence the effective RIR. The high-Mg calcite RIR was derived from coralline algae with an average MgCO₃ content of 11.3 wt%. Peak widening would increase with natural variability, while differences in means would shift peak locations. We did not see differences in location or shape between standards and the abalone samples, suggesting that the standards were sufficiently comparable for the purposes of RIR calibration.

Finally, each mineral standard was prepared as a single bulk batch prior to calibration. Although analytical replication was extensive across several levels of measurement, variability associated with independent preparation (e.g., cleaning or grinding effects) was not quantified and is therefore not represented in the uncertainty model. Incorporating multiple independently-prepared batches would allow preparation-level variability to be formally propagated through the calibration framework, but would have hindered our ability to identify other sources of variation.

Collectively, these considerations may reflect second-order sources of uncertainty relative to the primary effects underpinning the calibration, but also suggest that the errors we present are lower bounds. Within this scope, the framework provides a practical solution for skeletal carbonate analysis that is both accessible and quantifies uncertainty, while offering a possible foundation for extension to other mineralogical systems.

### Precision and accuracy of XRD analysis of biogenic carbonate mixtures

One approach to reducing estimation error is to average estimates from multiple independent subsamples. This approach is especially useful when estimation of individual points is critical; it is often less important when examining trends across multiple samples, as errors tend to cancel each other out. The error of an estimate is a combination of precision error and accuracy error, as measured by the standard error and the bias of an estimator. The relationship is often described by the root mean squared error, which is the square root of the sum of the squared standard error and squared bias. When *n* independent subsamples are averaged together, the standard error decreases with the square root of *n*, but there is no change to the bias. It is not uncommon in measurement systems for accuracy to be much better than precision, in which case it is reasonable to ignore bias when estimating overall error. For example, in Fig 4a, we observe that estimated and actual weight % align but that there is substantial variation around this alignment. However, bias can result from many factors including differences in sample preparation or machine drift between calibrations. When many independent subsamples are averaged together, precision error of the averaged values can reduce to the point where bias becomes the most important source of error. It is therefore useful to understand both components when determining the potential benefit of subsampling and averaging.

Estimates of mineral proportions from XRD analyses taken of the same sample were reasonably precise with a standard deviation of only 1 wt%. Sampling error was, naturally, greater at about 5 wt%. In other studies, error reported associated with XRD-based skeletal carbonate mineralogy is typically ±1 wt% [e.g., 30] This error is consistent with the values we have found for re-analysis of the same sample, but inconsistent with different samples of the same powder, where we found the sampling standard deviation to be ±5 wt% (see Fig 6), similar to the sampling errors observed in our analysis of test samples of 17 standard mixtures (see Fig 5).

When averaging 20 different samples together, the estimate for aragonite was 3% too high, LMC close to correct, and HMC 3% too low. Further, t-based confidence intervals based on the subsamples for aragonite and HMC did not include the correct values; this was true even when only 10 subsamples were averaged. However, the limits of the confidence intervals were within 1 wt% of the correct values (0.3 wt% for aragonite, 0.9 wt% for LMC).

We also examined the benefit of averaging across the 34 test samples for the standard mixtures (raw data available in [19]). The mean absolute error for the individual test samples was 2.2 wt%, which reduced to 1.5 wt% if estimates from the two related samples were averaged together. Combining all of this information suggests there is benefit to averaging when estimation of individual samples is important, so we recommend averaging up to five subsamples together, or explicitly including an accuracy component to the estimated error if averaging more subsamples.

Finally, we note that skeletal carbonate studies [e.g., 31] have typically reported mineralogy with more precision than this study suggests is justified, e.g., including decimal places. We recommend either explicitly stating standard errors, as in this study, or reporting in broad percentage categories without decimals, as in [30].

### Skeletal carbonate mineralogy of abalone

One of the reasons that we have heretofore lacked a system for estimating trimineral skeletal composition from XRD patterns is that we have not needed it. Almost all marine skeletal carbonates are monomineralic, and some are bimineralic. One Antarctic bivalve *Laternula elliptica* has been shown to precipitate calcite, aragonite and vaterite in part of its shell [32]. It was the observation of some tri-mineral patterns in *Haliotis iris*, suggesting that two Mg-carbonate minerals were present, that inspired this research and the development of our model. But how unusual is this composition among abalone?

Shell structure in abalone (genus *Haliotis*) is well known. Protoconch skeletons are mostly amorphous calcium carbonate [33], which in the veliger stage gradually crystallises into aragonite [34]. Juvenile shells are usually entirely aragonitic, but adult shells differentiate into two discrete layers, with an inner nacreous aragonitic layer overlain by a prismatic layer, which in turn lies under an organic periostracum which protects the shell from sea water. The outer prismatic layer is the most variable; it may be calcitic (e.g., *H. kamtschatkana, H. rufescens*), aragonitic (e.g., *H. asinina*, *H. glabra*), or both (*H. iris, H. rubra*). Table 3 reviews some of the variability in this genus. It has been suggested that mineralogical variation could be phylogenetic and could help with identification of the affinities of fossil abalone [35].

**Table 3:** Skeletal carbonate mineralogy of abalone in the genus *Haliotis* Linnaeus, 1758. Of the c. 60 species, 18 have been studied.

Abalone species	Locality	External (prismatic) shell layer	Internal (nacre) shell layer	References
*Haliotis asinina* Linnaeus, 1758	Australia, Komodo Island, Indian Ocean	Aragonite	Aragonite	[35–37]
*Haliotis discus hannai* Ino, 1953	China (farmed specimens)	Mixed aragonite and calcite	Aragonite	[33,38]
*Haliotis (Nordotis)* cf. *discus* Reeve, 1846	Japan	Mixed aragonite and calcite	Aragonite	[37]
*Haliotis fulgens* Philippi, 1845	S California, USA	Mixed aragonite and calcite	Aragonite	[33,37]
*Haliotis gigantea* Gmelin, 1791	Japan	Mixed aragonite and calcite	Aragonite	[36]
*Haliotis glabra* Gmelin, 1791	Java	Aragonite	Aragonite	[33,37]
*Haliotis iris* Gmelin, 1791	New Zealand	Mixed aragonite and calcite	Aragonite	[1,33,37,39]
*Haliotis kamtschatkana* Jonas, 1845	San Juan Is, Washington, USA	Calcite	Aragonite	[33,37]
*Haliotis laevigata* Donovan, 1808		Calcite	Aragonite	[39]
*Haliotis midae* Linnaeus, 1758		Calcite	Aragonite	[32]
*Haliotis pulcherrima* Gmelin, 1791		Aragonite	Aragonite	[33]
*Haliotis roei* Gray, 1826	prob. Australia	Mixed aragonite and calcite	Aragonite	[33,37]
*Haliotis rotundata (*nomen dubia according to Shepherd et al. 1995)	“unknown locality”	Mixed aragonite and calcite	Aragonite	[37]
*Haliotis rubra* Leach, 1814	Port Phillip Bay, Melbourne, Australia	Mixed aragonite and calcite	Aragonite	[40,41]
*Haliotis rufescens* Swainson, 1822	N California, USA, Chile	Calcite	Aragonite	[33,36,37,39,42]
*Haliotis spadicea* Donovan, 1808		Calcite	Aragonite	[33]
*Haliotis tuberculata* Linnaeus, 1758 and *H. tuberculata lamellosa* Lamarck, 1822	English Channel, France, Mediterranean (some reared in lab)	Protoconch ACC; Juveniles and young aragonite; Older adults mixed	Aragonite	[33,34,37,43–46]

The black-footed pāua *Haliotis iris* (Gmelin, 1791), in common with many abalone, has been known for some time to be bimineralic. Gray and Smith [1], for example, showed that the thickness of an external calcite layer relative to the internal aragonitic nacre was related to coastal hydrologic conditions. Small juvenile pāua raised in low-pH sea water had reduced calcite in their shells, which also showed signs of dissolution [47]. Recent analyses (as part of other studies) suggest that some adults and a few juveniles in this species form their shells with aragonite and both low-Mg calcite and high-Mg calcite – a composition not often reported in marine molluscs.

It is, of course, possible that the high-Mg calcite (HMC) detected could originate from the crustose coralline algae that commonly encrust pāua shells in the New Zealand subtidal zone. If so, we would expect to find no HMC in unencrusted shells, and we would expect the Mg content of the HMC to be similar to that of local coralline algae. Analyses of unencrusted specimens from aquaculture farms (sample numbers that begin HI-MB) do, however, find HMC in at least some samples. Mg-content in the HMC in *H. iris* is generally around 6–7 wt% MgCO_3_; New Zealand coralline algae precipitate skeletal carbonate in the range of 11–14 MgCO_3_ [48]. It thus appears that *H. iris* does indeed precipitate three discrete minerals in a single shell, uniquely among abalone and possibly molluscs.

### Factors influencing mineral proportions in *Haliotis iris*

Shell age explained 31% of the variation in aragonite (part *R*^2^), much more than sampling location (10%) or shell length (7%). Combined, the three predictors explained 46% of the variation in aragonite (conditional *R*^2^). Similar patterns of variation in LMC were explained as in aragonite: 47% overall, 29% by shell age, 16% by location, and 2% by length. For the HMC analysis, we only considered location as Kaikoura and Other. The model explained 78% of the variation in HMC given presence, primarily through sampling location being Kaikoura or not (70%) with shell age (7%) and length (8%) able to explain small amounts as well.

Abalone precipitate mainly LMC at the edge of the shell [49]. Over time, aragonite is added on the inner surface, while LMC wears off the outer surface. The progressive loss of LMC from the shell with age results from surface abrasion, damage, and dissolution over time, especially after the thin periostracum has been lost (see, e.g., [27]). Old specimens of *H. iris* are easily recognisable because the calcite external layer has worn off at the spire and the nacre shines through. It makes energetic sense to use relatively inexpensive LMC as a sacrificial layer over tougher yet more soluble aragonitic nacre. HMC is used opportunistically, in this case often in Kaikoura and rarely elsewhere. HMC appears to replace both aragonite and LMC when used, as evidenced by Kaikoura having lower average values for both.

## Importance and conclusions

*Haliotis iris* is taonga (treasured) in Aotearoa New Zealand as a traditional food, a cultural icon, a major export earner, and an essential component of Māori art. It is one of the largest wild-caught abalone fisheries in the world [27], not least because the shell is especially well-suited to craft and art, especially in jewelry [50]. While the intense blues and the strong iridescence appeal to artisans, the shell’s strength and hardness are equally important in ensuring that art works are resilient and fit-for-purpose. Shell quality is likely to be related to individual survival and thus to population stocks. Further, live pāua exports are costed based on weight, a considerable proportion of which is shell [49].

Our data show that *Haliotis iris* is special in yet another way – unusual among gastropod molluscs in precipitating high-Mg calcite, and also a trimineral mixture of aragonite, HMC and LMC. In the course of developing a method for measuring these three minerals using standard XRD patterns, we have created a model that is more accurate than previous empirical methods of determining mineralogy yet still simple to use compared to full pattern summation or Rietveld methods; this is also the first detailed analysis of precision in XRD-based skeletal carbonate mineralogy analysis.
